# Laser Gyro Temperature Compensation Using Modified RBFNN

**DOI:** 10.3390/s141018711

**Published:** 2014-10-09

**Authors:** Jicheng Ding, Jian Zhang, Weiquan Huang, Shuai Chen

**Affiliations:** College of Automation, Harbin Engineering University, Harbin 150001, China; E-Mails: zhangjian12@hrbeu.edu.cn (J.Z); huangweiquan@hrbeu.edu.cn (W.H.); chenshuai063@163.com (S.C.)

**Keywords:** laser gyro, temperature compensation, radial basis function neural network, Kohonen network, orthogonal least squares

## Abstract

To overcome the effect of temperature on laser gyro zero bias and to stabilize the laser gyro output, this study proposes a modified radial basis function neural network (RBFNN) based on a Kohonen network and an orthogonal least squares (OLS) algorithm. The modified method, which combines the pattern classification capability of the Kohonen network and the optimal choice capacity of OLS, avoids the random selection of RBFNN centers and improves the compensation accuracy of the RBFNN. It can quickly and accurately identify the effect of temperature on laser gyro zero bias. A number of comparable identification and compensation tests on a variety of temperature-changing situations are completed using the multiple linear regression (MLR), RBFNN and modified RBFNN methods. The test results based on several sets of gyro output in constant and changing temperature conditions demonstrate that the proposed method is able to overcome the effect of randomly selected RBFNN centers. The running time of the method is about 60 s shorter than that of traditional RBFNN under the same test conditions, which suggests that the calculations are reduced. Meanwhile, the compensated gyro output accuracy using the modified method is about 7.0 × 10^−4^ °/h; comparatively, the traditional RBFNN is about 9.0 × 10^−4^ °/h and the MLR is about 1.4 × 10^−3^ °/h.

## 1. Introduction

The stability of the output of a laser gyro, which is a high-precision optical angular rate sensor, directly affects the accuracy of the laser inertial navigation system (LINS) [1]. A laser gyro is usually operated in complex temperature-changing environments. Temperature changes affect the physical properties, geometry and gas flow field of the laser gyro. Such changes can also activate the anomalous dispersion effect of a medium, resulting in scale factor error and the zero bias of a laser gyro [2]. Numerous experiments have demonstrated that the laser gyro scale factor changes slightly under different temperature conditions, but the zero bias is known to be the most susceptible to this change [3,4]. In fact, the zero bias of laser gyro is closely related to the temperature of its operating environment and to temperature change rate; thus, addressing the negative effect of temperature and temperature change rate on the zero bias of laser gyro improves the accuracy of the LINS [5,6]. The traditional modeling of zero bias temperature compensation is widely used in the LINS as a relatively simple and inexpensive scheme for relieving the temperature effect [7,8]. A temperature compensation model based on multiple linear regression (MLR) [9] has been used to improve the traditional compensation effect. However, the model parameters of MLR have to be identified. A limited zero bias improvement is achieved as a result of the accuracy and timeliness of the identified parameters.

Many factors with complicated nonlinear characteristics can affect the temperature characteristics of laser gyros because of the working environment. A neural network possesses excellent approximation ability for complex nonlinear functions, and it is relatively similar to the nonlinear function model, except that the training of a neural network must meet specific requirements [10]. A back propagation neural network (BPNN) has improved identification accuracy significantly by accurately fitting the temperature characteristics of laser gyros [11,12]. Relative to that for the BPNN, the temperature compensation method for the radial basis function neural network (RBFNN) has achieved higher accuracy and speed [12–18]. In [12], a set of static test data from 25 °C to 55 °C had been collected. Some comparative results demonstrated that the bias decreased to 0.016 °/h from 0.029 °/h using the BPNN compensation method, meanwhile, 0.012 °/h was obtained using the RBFNN compensation method. In [14], the RBFNN could have a ten-fold improvement in accuracy for a digital closed-loop fiber optic gyroscope. As a result of structure and principle analysis, [18] held that the RBFNN was faster than BPNN; however, a specific numerical result was not reported. By using the least squares method and clustering techniques to train the RBFNN and the simplified network structure, a significantly small compensation error was demonstrated in [19–21]. However, the RBFNN centers have to be randomly selected [22]. If the number of the RBFNN centers is too large, then heavy calculations have to be endured. This requirement degrades the efficiency of the algorithm in the process of forward selection. Meanwhile, if the number of the RBFNN centers is too small, then a numerical ill-conditioned problem emerges and leads to the poor generalization ability of the model, because the training set cannot objectively summarize the statistical characteristics of the overall sample.

The structure of the paper is as follows. In Section 2, the RBFNN and the Kohonen network are described. In Section 3, a modified RBFNN algorithm based on the Kohonen network and orthogonal least squares (OLS) is proposed for laser gyro temperature compensation. First, the algorithm classifies the randomly selected RBFNN centers preliminarily using the pattern classification feature of the Kohonen network. Second, the classified RBFNN centers and the OLS algorithm are applied to get a sorted hidden layer output vector. Third, the sorted vector is applied to train the RBFNN. Consequently, a modified RBFNN model is obtained. These steps are performed to avoid heavy calculations caused by the random selection of the RBFNN centers. A realizable flow chart of laser gyro temperature compensation is also presented in this section. In Section 4, a variety of comparable temperature compensation tests using different methods are designed. Interesting results and analyses are also provided in this section. In Section 5, conclusions are provided.

## 2. Structure Description of the RBFNN and the Kohonen Network

### 2.1. Structure of the RBFNN

The RBFNN is typically a three-layer forward network. It includes an input layer, a hidden layer and an output layer. Each layer serves a different function [23].

The first layer is the input layer. It consists of a number of source nodes (perception neurons) connected with the external environment. The second layer is the hidden layer comprising several hidden nodes. Only one hidden layer exists in the RBFNN, because of the output characteristics of the RBFNN. The third layer is the output layer, which responds to the input layer. It comprises some output nodes in the form of a linear summation unit. The nodes of the input layer transfer input signals to the hidden layer. The transfer from the input layer space to the hidden layer is nonlinear, whereas that from the hidden layer to the output layer space is linear [24,25]. The output nodes in the network calculate a linear combination of basic functions given by the output layer. The weights between the input layer and the hidden layer are known to be constant (one in this case); thus, only the weights between the hidden layer and the output layer are adjustable [26].

**Figure 1.**
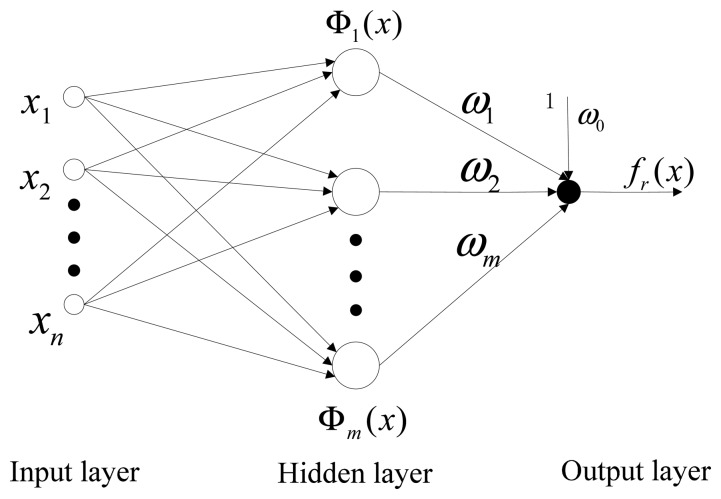
Typical structure of the radial basis function neural network (RBFNN).

The structure of the RBFNN with *n* inputs and one output is shown in Figure 1. The numbers of nodes for the three layers are *n*, *m* and one, respectively. *x*_1_, *x*_2_,…, *x_n_* are the first, second,…, and *n*-th input, which compose one input vector. *ω*_1_, *ω*_2_,…, *ω_m_* represent the weights between the hidden layer and the output layer. These weights are adjustable.

This network realizes the mapping *f_r_*: *R^n^*→*R*.

(1) $$f_{r}(x)=\omega_{0}+\sum_{i=1}^{m}\omega_{i}\Phi (∥x-c_{i}∥)$$

where *x* ∈ *R^n^* is the input vector, **Φ** (•) is the nonlinear transformation function, *ω_i_* (*i* = 1,2, ⋯,*m*) are the weights (or parameters), *c_i_* ∈ *R^n^* (*i* = 1,2, ⋯,*m*) are the centers of the neurons used in nonlinear transformation function of RBFNN, with the same dimensions as *x*, and *m* is the number of the centers.

The structure of the RBFNN in Figure 1 shows that the neuron weights are set between the input layer and the hidden layer. The center of each neuron and the local receptive field in the hidden layer determine the location and width of the RBFNN. The hidden layer in the RBFNN is weighted and superimposed by neurons in the output layer. As long as the number of neurons in the hidden layer is adequate and the selected centers, local receptive field and weights are appropriate, the RBFNN can fit any function with accuracy. In this study, a Gaussian function is selected as a nonlinear transform function Φ(**·**) for the RBFNN [27]. This function is given by:

(2) $$\Phi (u)=\text{exp}(-\frac{u^{2}}{\delta^{2}})$$

where *δ* is the local receptive field, which determines the width of the nonlinear transformation function surrounding the central points.

### 2.2. Structure of the Kohonen Network

**Figure 2.**
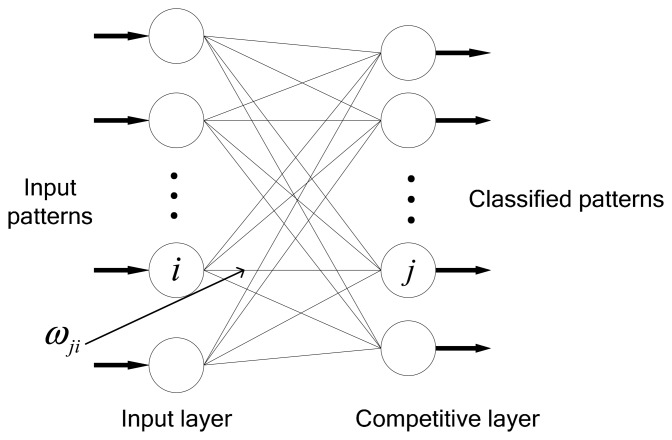
Structure of the Kohonen network.

The Kohonen network is a self-organizing competitive neural network based on stimulation of outside information to the cerebral cortex. When the brain receives specific temporal and spatial information outside through the senses, particular areas of the cortex are excited. Additionally, the mappings to the similar information from outside world are continuous [28]. The Kohonen network classifies the input set by adjusting the weights of the neurons. More specifically, the Kohonen network is a neural network procedure in which a layer of neurons is initialized with random weights and subsequently organized by inspection of the data to be classified. The organization procedure uses progressive adjustment of weights based on data characteristics and lateral interaction, such that the neurons with similar weights will tend to spatially cluster in the neuron layer. In this way, the irregular inputs can be classified automatically. A similar weight distribution and probability density distribution for input samples are obtained in the process of weight adjustment. The basic Kohonen network has a simple two-layer structure comprising an input layer and a competitive layer [29]. As shown in Figure 2, the competitive layer is also known as the output layer. A weight vector is associated with each neuron. Each output neuron is connected to the input layer via that weight vector. The dimensionality of the weight vector of each output neuron is the same as the dimensionality of the input vector. These weight vectors of the output neurons are compared with the input vectors, in order to determine the degree of activation of that neuron. This kind of connection enables the Kohonen network to learn “competitively,” which means that the neurons in the competitive layer compete for the classification of input patterns. During training, the output neuron that provides the highest activation to a given input pattern is declared the winner and is moved closer to the input pattern, whereas the remaining neurons are left unchanged.

The competition rules are defined as follows:

(3) $$x_{j}^{c}=\{\begin{array}{l} 1,S_{j}>\max(S_{l},l\neq j,l=1,2,\cdots n)\\ 0,\text{others} \end{array}$$

where $x_{j}^{c}$ represents the output state of the *j*-th neuron in the competitive layer. *S_j_* is the dot product of weights vector of the *j*-th neuron in output layer and input vector. A maximum *S_j_* means that the Euclidean distance between the weight vector and input vector is closest.

For a single input in the Kohonen network, the weights are updated after the winning neuron is identified. The weight of the winning neuron is increased to generate a large sum of neuron inputs when the input pattern appears again. The weight update rule is as follows:

(4) $$\Delta \omega_{ji}=\eta (x_{i}-\omega_{ji})$$

where *η* is a learning factor that represents the update rate of weights.

## 3. Modified RBFNN and Temperature Compensation Model for Laser Gyro

As mentioned previously, the RBFNN centers, local receptive field and weights of output neurons are the most important parameters in RBFNN training [30,31]. The RBFNN suffers from a large network scale and the numerical ill-conditioned problem when randomly selecting its centers. In the present work, first the gyro outputs are provided to the Kohonen network and are classified according to adjusted weights. Thus, each class of data is processed to get an input with the same statistical properties for the RBFNN. Then, as an optimal searching strategy, the OLS algorithm is used to calculate independently the contribution of the regression operator to the expected model, and a sorted hidden layer output vector is obtained. The OLS algorithm would be able to improve the RBFNN training efficiency.

The modeling steps for laser gyro temperature compensation based on the modified RBFNN are designed as follows:

Step 1: Conform the sample space by preprocessing the laser gyro zero bias and the temperature data.
Step 2: Process the sample data using the Kohonen network and record the classified data. The learning steps of the Kohonen network are as follows:
  (1) Randomly initialize the weights *ω_ij_* between the input layer and the RBFNN, and set the initial learning rate *η* (0). Here, *i* = 1,…, *n*; *j* = 1,…, *m*; *n* represents the number of nodes in the input layer of the Kohonen network and *m* represents the number of nodes in the input layer of the RBFNN.
  (2) *x*(*k*) represents the *n*-dimensional input vector at time *k*. Let *x*(*k*) be the input of the Kohonen network. The *k* is discrete sampling time.
  (3) Substitute *x*(*k*) into Equation (5), and calculate the distance *d_j_* of each output node.
    (5) $$d_{j}=\sum_{i=1}^{n}{\left(x_{i}(k)-\omega_{ij}(k)\right)}^{2},j=1,\cdot \cdot \cdot ,m$$
    where *ω_ij_*(*k*) represents the weight at time *k*.
  (4) Choose node j* with minimum distance *d_j_* as the winning output node.
  (5) Update the weight of node j* according to the following equation:
    (6) $$\omega_{ij}(k+1)=\omega_{ij}(k)+\eta (k)\cdot (x_{i}(k)-\omega_{ij}(k))$$
    where *η*(*k*) represents the learning rate at time *t*, which decreases with time; *ω_ij_*(*k* + 1) represents the weight at time *k* + 1.
  (6) If the variation of *ω_ij_* is equal to zero or is considerably small, then the learning process is completed. Otherwise, proceed to Step (2).
Step 3: Preprocess the data classified by the Kohonen network, and let the preprocessed data be the RBFNN centers. Then, sorted hidden layer output *P* and sorted RBFNN centers are obtained using the OLS algorithm. The main steps are as follows:
  (1) Choose Φ(*x*) = exp(*x*^2^/*δ*^2^) as the transform function of the RBFNN and set the local receptive field *δ*. Then, set the maximum number *N* of network training and the threshold *ρ*, and set the preprocessed data as the RBFNN centers.
  (2) Obtain the hidden layer output vector *P*(*P* ⊆ ℜ*^Q^*^×^*^Q^*) through the transform function. Then, the steps for getting sorted *P* using the OLS algorithm are as follows (as an accessory, sorted RBFNN centers have been also obtained):
    Step (1): When *k* = 1, for 1 ≤ *I* ≤ *Q*, set:
      (7) $$b_{1}^{\left(i\right)}=P_{i}$$
      Calculate the error reduction rate for the *i*-th center as:
      (8) $${\left[\text{err}\right]}_{1}^{i}=\frac{{\left(b_{1}^{(i)T}y_{d}\right)}^{2}}{b_{1}^{(i)T}b_{1}^{\left(i\right)}\cdot y_{d}^{T}y_{d}}$$
      Search the maximum of the error reduction rate using the following equation:
      (9) $${\left[\text{err}\right]}_{1}^{i1}=\max\left\{{\left[\text{err}\right]}_{1}^{i},1\leq i\leq Q\right\}$$
      Select:
      (10) $$b_{1}=P_{i1}$$
      and set the center as *c*_1_ = *c_i_*_1_.
    Step (*k*): If *k* ≥ 2, for 1 ≤ *i* ≤ *Q*, *i* ≠ *i*_1_,…, *i* ≠ *i_k_*_−1_, then calculate:
      (11) $$a_{jk}^{\left(i\right)}=\frac{b_{j}^{T}P_{i}}{b_{j}^{T}b_{j}}\;1\leq j\leq k-1$$
      Set:
      (12) $$b_{k}^{\left(i\right)}=P_{i}-\sum_{j=1}^{k-1}a_{jk}^{\left(i\right)}b_{j}$$
      Calculate:
      (13) $${\left[\text{err}\right]}_{1}^{\left(i\right)}=\frac{{\left(b_{k}^{(i)T}y_{d}\right)}^{2}}{b_{k}^{(i)T}b_{k}^{\left(i\right)}\cdot y_{d}^{T}y_{d}}$$
      Search and obtain:
      (14) $${\left[\text{err}\right]}_{k}^{ik}=\max\left\{{\left[\text{err}\right]}_{k}^{i},1\leq i\leq Q,i\neq i_{1},i\neq i_{2},\cdot \cdot \cdot ,i\neq i_{k-1}\right\}$$
      Select:
      (15) $$b_{k}=b_{k}^{\left(i_{k}\right)}$$
      and set *c_k_* = *c_ik_*.
    Step (*k*+1): Repeat Step (*k*) until:
      (16) $$1-\sum_{j=1}^{N_{1}}{\left[\text{err}\right]}_{j}<\rho$$
      Stop at Step *N*_1_.
Step 4: Complete the RBFNN training using the sorted hidden layer output *P* and obtain an expected laser gyro temperature compensation model.

**Figure 3.**
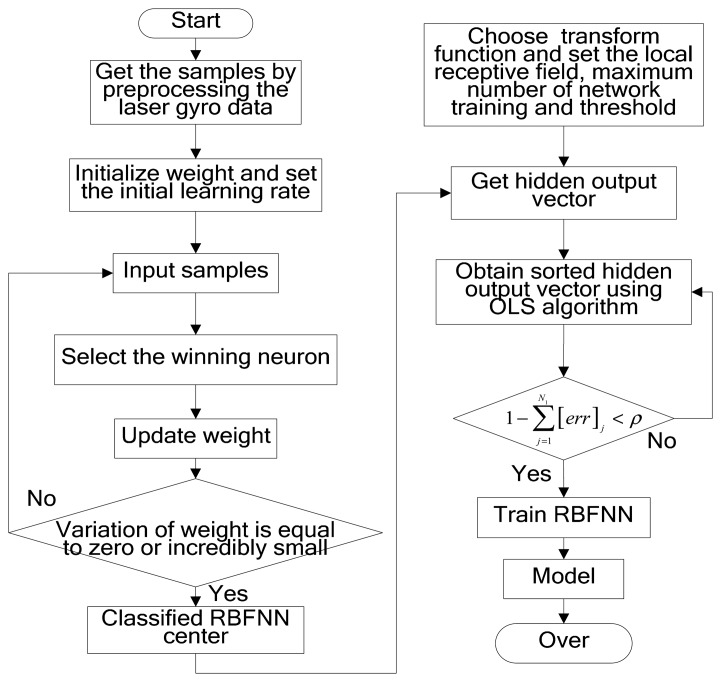
Flow chart of modified RBFNN modeling. OLS, orthogonal least squares.

The flow chart of the modified RBFNN modeling is shown in Figure 3. The inputs for the designed laser gyro temperature compensation experiment are composed of the gyro temperature *T* and the temperature change rate d*T*/d*t*. The temperature change rate is calculated by the temperature. The bias of the laser gyro is the desired output *y_d_*. In the main process of modeling for the modified RBFNN laser gyro temperature compensation, the samples are classified by the Kohonen network, and a set of new data is obtained. The new data are classified RBFNN centers. Then, a sorted hidden layer output vector *P* is carried out and applied to train the RBFNN; as a training result, the modified RBFNN model for laser gyro zero bias temperature compensation would be obtained.

## 4. Laser Gyro Zero Bias Temperature Compensation Test and Analysis

### 4.1. Laser Gyro Data Acquisition and Preprocessing

To achieve a comprehensive assessment of the proposed temperature compensation scheme, the following three experiments were set up for obtaining the typical test data. The main performance parameters of the used laser gyro are as follows: bias stability is 0.008 °/h; the dynamic range is ±400 °/s; the bandwidth is 800 Hz; the resolution is less than 0.001°; and the operating temperature range is from −54 °C to 85 °C. The zero bias of the laser gyro and the temperature data were collected under six kinds of environments, as well as under normal temperature, low temperature and high temperature; the temperature changing rates were 1 °C/min, 3 °C/min and 5 °C/min. The experiments were performed twice and obtained two data sets, each of which comprised six groups of data. The two data sets were used in modeling and examination. In the experiments, an inertial measurement unit (IMU) with three laser gyros was in a static state, and the three orthogonal laser gyros pointed to the east, to the north and upward. The outputs of the east laser gyro were zero biased, because of the zero component of the Earth rotating in the east.

Normal temperature data acquisition: The laser gyro was placed in a large temperature-controlled cabinet, and the constant temperature was set to 20 °C. The laser gyro was then powered and kept on for one hour. The laser gyro outputs and the changes in temperature were recorded. The sampling frequency was 100 Hz. Eight-hour laser gyro outputs and temperature data were acquired.

r array exposed to different odors/gases. acquisition: The temperatures in the temperature-controlled cabinet were set to −40 °C and 60 °C for the low temperature and high temperature data acquisition, respectively. The other steps were the same as those in the normal temperature data collection.

Variable temperature data collection: the range of temperature was controlled between −40 °C and 60 °C. The initial temperature of 0 °C was maintained for one hour. The laser gyro was then powered on after setting the temperature change rate to 1 °C/min. The laser gyro outputs and temperature data were recorded. When the temperature reached 60 °C, it was maintained for two hours, after which the temperature started to decrease by 1 °C/min. When the temperature reached −40 °C, it was maintained for two hours, after which the temperature rose by 1 °C/min. The process above was repeated, and eight-hour data were obtained by a 100-Hz sampling frequency. In the same way, the laser gyro and temperature data were collected when the temperature change rates were 3 °C/min and 5 °C/min.

**Figure 4.**
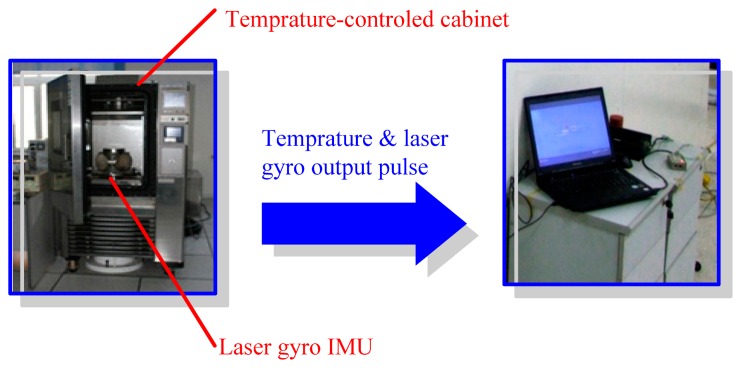
Data collection experiment setup.

Equipment was set up (Figure 4) to collect the above data. The temperature-controlled cabinet and the temperature sensor in the laser gyro provided the temperature parameters. The temperature in the temperature-controlled cabinet was controlled by a specific software. The temperature parameters and the output pulses of the laser gyro were collected.

**Figure 5.**
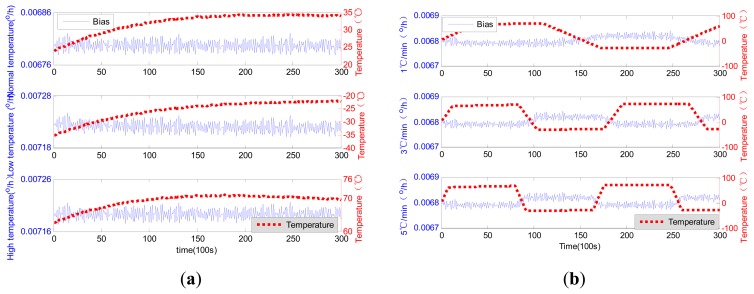
Filtered zero bias and for the laser gyro: (**a**) constant temperature; (**b**) variable temperature.

The laser gyro outputs acquired using the above equipment were not likely to change with the temperature, because of the presence of high-frequency noise. The outputs should be preprocessed by a low-pass filter before temperature compensation. The low-pass filter was designed for a simple averaging filter with 100 s; meanwhile, the temperature data were also filtered using the filter. Filtered laser gyro outputs with temperature changes are shown in Figure 5. In the figure, the scale of the horizontal axis is set to 100 s. The succeeding figures use the same scale.

In Figure 5, the zero bias is relatively stable under constant temperature conditions, with the average zero bias at 20 °C being 6.796 × 10^−3^ °/h. Compared with that under normal temperature conditions (20 °C), the values of the average zero bias under the high and low temperature conditions are 7.218 × 10^−3^ °/h and 7.194 × 10^−3^ °/h, respectively, both of which are greater than that under 20 °C.

The stability of the laser gyro zero bias weakens when temperatures are not constant and, instead, continuously change. The change trends with temperature are obvious and considerably unstable under quick temperature changes. In sum, both temperature and temperature change rate affect the accuracy of the laser gyro output, especially the zero bias.

### 4.2. Temperature Compensation Results and Analysis for Laser Gyro Zero Bias

The modeling procedure for laser gyro zero bias using the proposed temperature compensation scheme was designed as a flow chart (Figure 3). For comparison purposes, the MLR and the traditional RBFNN were also employed in this work.

The MLR model was used in selecting regression variables using approximate Bayesian information criteria to eliminate unimportant variables in the compensation model. When the criterion function achieves the smallest value, the selected variables suggest the best compensation model. In this work, the best model comprised a constant term, temperature *T*, and temperature changing rate d*T*/d*t*. The coefficients of the selected regression variables were calculated, and the model was verified. The detailed modeling steps using MLR are available in [9].

For the traditional RBFNN scheme, the value of *ρ* in Equation (16) should be between zero and one, and the sum of errors should be equal to 3.0 × 10^−4^. These parameters must be considered because of the accuracy of the laser gyro and the root mean square of the sum of target errors, which was equal to the standard deviation of the laser gyro zero bias. The neuron numbers of the traditional RBFNN training were 108, 112, 115, 121, 129 and 135 for the six-group data. The final model for laser gyro zero bias could finally be obtained.

**Figure 6.**
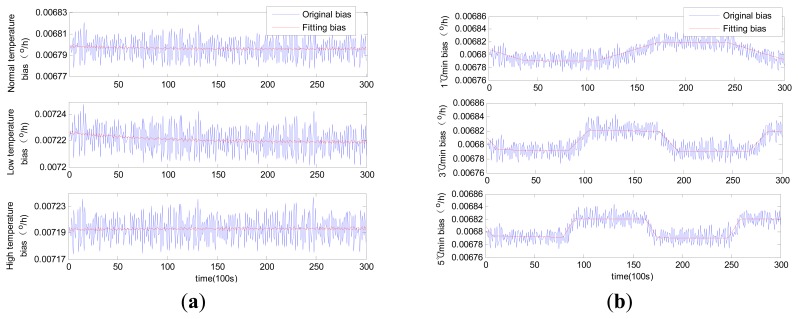
Forecasting curves using multiple linear regression (MLR): (**a**) constant temperature; (**b**) variable temperature.

**Figure 7.**
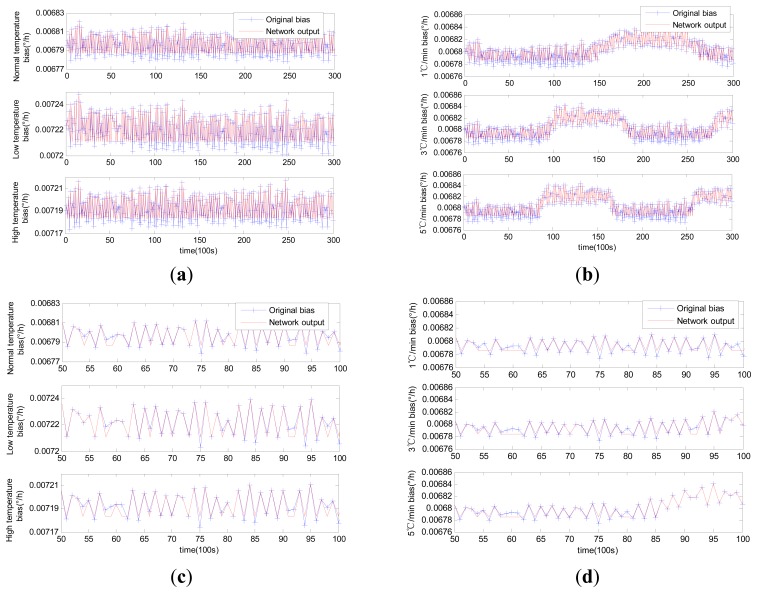
Identification curves using traditional RBFNN: (**a**) constant temperature; (**b**) variable temperature; (**c**) enlarged (a) in the horizontal axis from 50 to 100; (**d**) enlarged (b) in the horizontal axis from 50 to 100.

**Figure 8.**
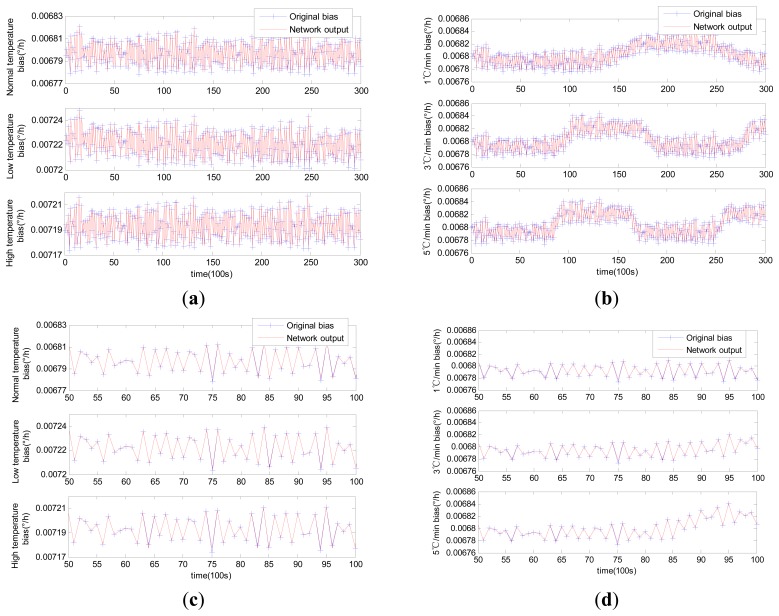
Identification curves using modified RBFNN: (**a**) constant temperature; (**b**) variable temperature; (**c**) enlarged (a) in the horizontal axis from 50 to 100; (**d**) enlarged (b) in the horizontal axis from 50 to 100.

Comparable identification results using MLR, traditional RBFNN and modified RBFNN are shown in Figures 6, 7 and 8.

Although MLR successfully tracked the trend of the laser gyro zero bias, it was unable to completely describe the zero bias. Such failure would naturally affect the compensation results to a certain extent. This condition is attributable to the fact that influential factors were ignored in the selection of regression variables in MLR modeling. The training of the neural network is quite different from that of MLR. The neural network trains the model by updating the weights instead of confirming the coefficients through select regression variables. The forecasting results of both the traditional RBFNN and the modified RBFNN are available. Note that the forecasting curve obtained through the traditional RBFNN model cannot properly track the zero bias. The modified RBFNN has already achieved accurate results under different constant temperatures and temperature change rates. This phenomenon is attributable to the classified centers obtained from the Kohonen network; because the Kohonen network is able to generalize data characteristics instead of randomly selecting RBFNN centers.

**Figure 9.**
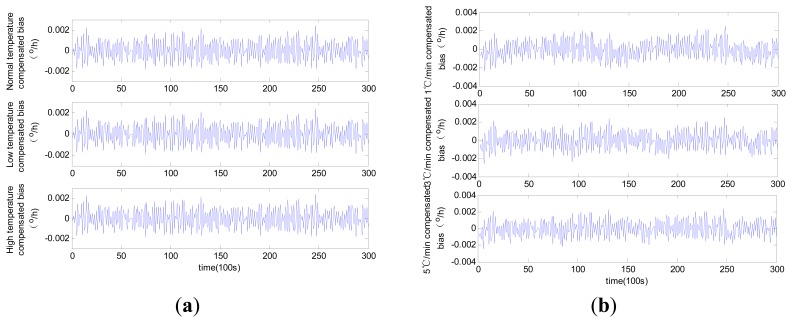
Compensated zero bias history using MLR: (**a**) constant temperature; (**b**) variable temperature.

**Figure 10.**
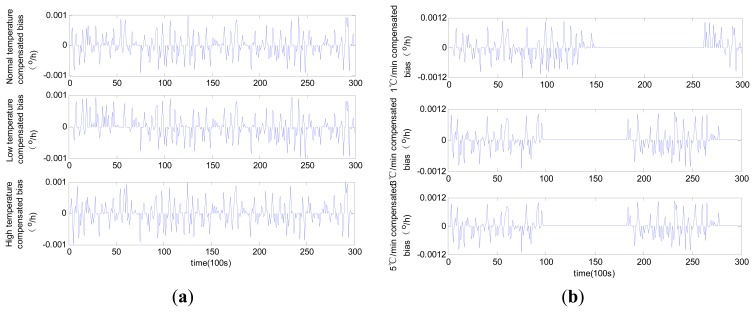
Compensated zero bias history using traditional RBFNN: (**a**) constant temperature; (**b**) variable temperature.

**Figure 11.**
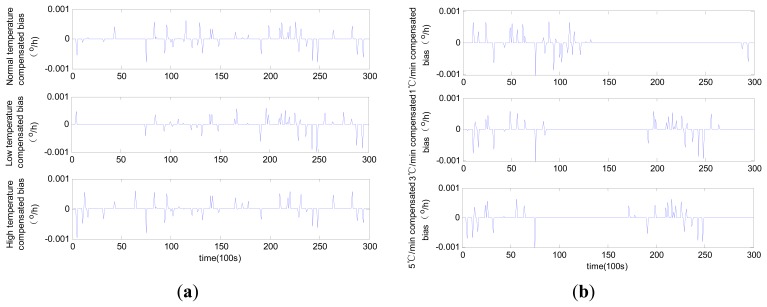
Compensated zero bias history using modified RBFNN: (**a**) constant temperature; (**b**) variable temperature.

The compensated laser gyro zero biases obtained using the three different methods are shown in Figures 9, 10 and 11.

The results suggest that all the compensated results for zero bias are not affected by temperature change trends. The different levels of accuracy for the compensated laser gyro outputs result from the different identification abilities of the three methods. The MLR method shows a relatively lower performance than the other two methods. Given the same number of training samples, the modified RBFNN method achieves more accurate laser gyro output compared with the traditional RBFNN. The detailed comparison of the three methods based on the test results is shown in Table 1.

In Table 1, the constant temperatures −40 °C, 20 °C and 60 °C represent the low temperature, normal temperature and high temperature condition, respectively. As shown in the table, the compensated laser gyro produces higher accurate outputs. The compensated outputs accuracies with MLR method are improved to 1.4 × 10^−3^ °/h from 7 × 10^−3^ °/h under all temperature conditions. This result is due to the estimated parameters accuracy in the MLR model. The compensated laser gyro outputs of both the RBFNN and the modified RBFNN reach 10^−4^ °/h within the entire range of temperature change. However, the compensation error of the MLR method is slightly larger than those of the other two methods. The MLR, traditional RBFNN and modified RBFNN methods show five-fold, eight-fold and ten-fold improvements in accuracy, respectively.

Before compensation, the accuracy of the outputs in normal temperature is higher than that in low or high temperature conditions, and the accuracy of the outputs under variable temperatures is slightly lower than that under constant temperatures. A high temperature change rate equates to low accuracy. However, this phenomenon is not obvious after compensation using all three methods. For instance, the output accuracies are 7.218 × 10^−3^ °/h, 6.796 × 10^−3^ °/h and 7.194 × 10^−3^ °/h under normal, low and high temperature conditions, respectively, and 6.918 × 10^−4^ °/h, 6.922 × 10^−4^ °/h and 6.986 × 10^−4^ °/h, respectively, after compensation with the modified RBFNN model. According to the changes of bias, we can conclude that the modified RBFNN model can ideally avoid the effect of temperature and temperature change.

The compensation results of the modified RBFNN and the traditional RBFNN are highly accurate, with the former being higher than the latter. Under any type of tested environment, the accuracy of the laser gyro can reach 7.0 × 10^−4^ °/h after compensation using the modified RBFNN. The running time of the modified RBFNN for all the tested data is less than 83 s, which is lower than that of the traditional RBFNN. Hence, the modified RBFNN is more effective than the traditional method.

## 5. Conclusions

Given their compensation accuracy, the MLR and the traditional RBFNN compensation method require large calculations and result in poor generalization in the random selection of the RBFNN centers. A modified RBFNN method based on OLS and the Kohonen network is thus proposed to complete high-precision laser gyro zero bias temperature compensation. The new compensation principle and the modeling steps for the proposed method are introduced in this paper in detail. Tests and analyses within an entire range of temperature change are completed using the three methods. Six kinds of temperature environments, normal temperature, low temperature, high temperature and varying temperature with temperature changing rates of 1 °C/min, 3 °C/min and 5 °C/min, are considered and verified accordingly. A database of the models is then built, and different models are selected according to the temperature sensor outputs for specific running environments. The analysis results show that the proposed temperature compensation scheme is more stable and achieves higher accuracy and efficiency compared with the other two methods. The accuracy of the compensated laser gyro output can reach 7.0 × 10^−4^ °/h, which represents a ten-fold improvement in accuracy. Widely used adaptability tests, such as that for low-cost MEMS gyro, and navigation accuracy improvement for a final inertial navigation system will be studied further.

## Figures and Tables

**Table 1.** Comparison of compensation results of the three methods.

**Constant Temperature**		**−40 °C**	**20 °C**	**60 °C**
**Before Compensation σ(°/h) (×10^−3^)**		7.218	6.796	7.194
**After Compensation σ(°/h) (×10^−4^)**	MLR	13.953	13.953	13.954
	RBFNN	8.93	8.944	8.93
	Modified RBFNN	6.918	6.922	6.926
$\text{Accuracy Improvement}(\frac{\text{Before compensation}}{\text{After compensation}})$	MLR	5.17	4.87	5.15
	RBFNN	8.08	7.60	8.05
	Modified RBFNN	10.43	9.82	10.39
**Running Time (s)**	MLR	—	—	—
	RBFNN	140.8	142.4	141.3
	Modified RBFNN	79.5	82.6	81.7
**Variable Temperature**		**1 °C/min**	**3 °C/min**	**5 °C/min**
**Before Compensation σ(°/h) (×10^−3^)**		6.804	6.804	6.806
**After Compensation σ(°/h) (×10^−4^)**	MLR	14.276	14.180	14.122
	RBFNN	8.916	8.928	8.944
	Modified RBFNN	6.930	6.922	6.912
$\text{Accuracy Improvement}(\frac{\text{Before compensation}}{\text{After compensation}})$	MLR	4.77	4.80	4.82
	RBFNN	7.63	7.62	7.61
	Modified RBFNN	9.818	9.832	9.847
**Running Time (s)**	MLR	—	—	—
	RBFNN	141.6	140.2	139.8
	Modified RBFNN	80.4	82.6	80.3
